# Can *Patrinia villosa* water extract be taken during FOLFOX treatment in colon cancer? – evidences from a proof-of-concept preclinical study

**DOI:** 10.3389/fphar.2026.1756234

**Published:** 2026-04-01

**Authors:** Grace Gar-Lee Yue, Kit-Man Lau, Dandan Hu, Ka-Ki Yuen, Julia Kin-Ming Lee, Si Gao, Xiaotong Lu, Simon Siu-Man Ng, Zhong Zuo, Kam Wa Chan, Clara Bik-San Lau

**Affiliations:** 1 Department of Pharmacology and Pharmacy, Li Ka Shing Faculty of Medicine, The University of Hong Kong, Hong Kong, Hong Kong SAR, China; 2 Institute of Chinese Medicine, The Chinese University of Hong Kong, Hong Kong, Hong Kong SAR, China; 3 Department of Surgery, The Chinese University of Hong Kong, Hong Kong, Hong Kong SAR, China; 4 School of Pharmacy, The Chinese University of Hong Kong, Hong Kong, Hong Kong SAR, China; 5 School of Chinese Medicine, Hong Kong Baptist University, Hong Kong, Hong Kong SAR, China; 6 Vincent V.C. Woo Chinese Medicine Clinical Research Institute, School of Chinese Medicine, Hong Kong Baptist University, Hong Kong, Hong Kong SAR, China; 7 School of Chinese Medicine, Li Ka Shing Faculty of Medicine, The University of Hong Kong, Hong Kong, Hong Kong SAR, China

**Keywords:** 5-fluorouracil, dihydropyrimidine dehydrogenase, FOLFOX, folinic acid, oxaliplatin, Patrinia villosa, serine hydroxymethyltransferase

## Abstract

**Background:**

5-fluorouracil-based regimen FOLFOX (5-fluorouracil, folinic acid, oxaliplatin) is the first-line chemotherapeutic for metastatic colorectal cancer patients. On the other hand, the medicinal herb *Patrinia villosa* (Thunb.) Dufr. [Caprifoliaceae] is commonly prescribed by Chinese medicine practitioners to colorectal cancer patients in recent years. Thus, the possibility of concurrent use of *P. villosa* with FOLFOX occurs. The present study aimed to investigate whether *P. villosa* water extract would interfere with the metabolism and anti-tumor efficacy of chemotherapy regimen FOLFOX in preclinical colon cancer models.

**Methods:**

Human HCT-116 xenograft-bearing and murine Colon-26 orthotopic tumor-bearing mouse models were established and received FOLFOX and/or PV water extract treatments for 3 weeks. FOLFOX was administered intravenously once a week and PV water extract was administered daily by oral gavage except on the FOLFOX administration day. The levels of metabolising enzymes dihydropyrimidine dehydrogenase (for 5-fluorouracil), serine hydroxymethyltransferase (for folinic acid), as well as molecules, cysteine, methionine and reduced glutathione, involved in oxaliplatin biotransformation, were determined in plasma of xenograft-bearing mice receiving FOLFOX and/or PV water extract treatment. The tumor progression, metastasis and tumor microenvironment in mice were also assessed.

**Results:**

The PV water extract treatment did not affect the plasma levels of metabolising enzymes of 5-fluorouracil and folinic acid, nor the plasma levels of cysteine, methionine and reduced glutathione in HCT-116 xenograft-bearing mice. On the other hand, the combined treatment of FOLFOX and PV water extract inhibited both HCT-116 and Colon-26 tumor growth, with HCT-116 tumor weight of FOLFOX plus PV water extract group being the lowest among all treated groups. PV water extract treatment could further potentiate the anti-angiogenesis, apoptosis and immunomodulatory effect of FOLFOX in colon tumors.

**Conclusion:**

This is the first report to demonstrate that no observable interference induced by PV water extract on the plasma levels of selected metabolism enzymes and precursor molecules of FOLFOX component drugs in colon tumor-bearing mice. The combined use of FOLFOX plus PV water extract resulted in better anti-tumor efficacy in colon cancer mouse models than single treatment alone. The beneficial potentials of combined use of FOLFOX and PV water extract has been scientifically verified, which supports the clinical use in colon cancer management.

## 1 Introduction

Colorectal cancer (CRC) is one of the most frequent malignancies with high incidence worldwide (Bray et al., 2024). At present, 5-fluorouracil (5-FU)-based regimens such as FOLFOX, FOLFIRI and FOLFOXIRI are considered to be the first-line therapeutic modalities for metastatic CRC patients (Cervantes et al., 2023). FOLFOX regimen is a combination of 5-FU, folinic acid (also known as leucovorin) and oxaliplatin. Owing to varied patients’ response rate to FOLFOX (estimated at around 50%) (Pourghasemian et al., 2020) and the occurrence of notable 5-FU-induced adverse effects, there is a persistent need to search for alternative treatment, such as herbal medicines, as adjuvant therapy on CRC to allow possible FOLFOX regimen dosage reduction to decrease side effects. Nevertheless, the potential interference of herbal medicines on FOLFOX treatment would be the major concerns of oncologists, clinicians and cancer patients.

In the presence of thymidine phosphorylase and thymidine kinase, 5-FU is converted to fluorodeoxyuridine monophosphate (FdUMP) which in turn forms a ternary complex with thymidylate synthase and 5,10-methylene tetrahydrofolate (5,10-MTHF). Folinic acid potentiates the activity of 5-FU by stabilizing the complex via the augmentation of 5,10-MTHF concentration (Deyme et al., 2019). Binding of thymidylate synthase to the complex inhibits thymidine formation and consequently DNA synthesis. On the other hand, 5-FU is anabolized by orotate phosphoribosyl transferase to 5-fluorouridine triphosphate (FUTP) which incorporates into RNA as a fraudulent base to interfere RNA synthesis (Miura et al., 2010). Oxaliplatin, a platinum-based chemotherapeutic agent with a 1,2-diaminocyclohexane (DACH) carrier ligand, induces the formation of DNA bis-adducts, thus impeding DNA replication and resulting in cell cycle arrest or apoptosis (Di Francesco et al., 2002). It was proposed that the mechanism for FOLFOX synergism involved the suppression of ATPase, the over-expression of glutathione exporters and the decrease of glutathione levels by oxaliplatin metabolite oxalate, which were all mediated by 5-FU (Theile et al., 2009).

Despite possessing prominent clinical efficacy in stage II or III colon cancer (Andre et al., 2009), 5-FU-based regimens are often coupled with side effects such as diarrhea, alopecia, anemia, leucopenia and peripheral neuropathy. The 5-FU-associated toxicities are aggravated or even lethal in patients who have dihydropyrimidine dehydrogenase deficiency (Johnson and Diasio, 2001; Dhelens et al., 2016). Dihydropyrimidine dehydrogenase (DPD) is the initial and rate-limiting enzyme responsible for the catabolism of 5-FU. After administered into human body, only 1%–3% of 5-FU is activated to cytotoxic metabolites such as FdUMP and FUTP, whilst more than 80% of 5-FU undergoes rapid degradation to dihydrofluorouracil (FUH_2_), an inactive metabolite, by DPD (Mattison et al., 2002). Therefore, DPD plays a vital role in determining a patient’s response to 5-FU. A reduced activity of DPD results in a substantially lengthened half-life of 5-FU and an increased risk of severe toxicity (van Kuilenburg et al., 2012; Sharma et al., 2019). With regards to the conversion of folinic acid into its active form, 5,10-MTHF, the reaction is catalyzed by the vitamin B6-dependent serine hydroxymethyltransferase (SHMT), with the simultaneous conversion of serine into glycine (Ducker et al., 2017). Hence, the enzymes DPD and SHMT play crucial role in the metabolism of 5-FU and folinic acid, respectively. On the other hand, oxaliplatin undergoes rapid and non-enzymatic biotransformation to form monochloro-, dichloro- or diaquo-DACH that interferes with DNA synthesis. However, in the presence of cysteine, methionine or reduced glutathione, oxaliplatin is converted into chemically unreactive species (Di Francesco et al., 2002).

Herbal medicines are frequently consumed by cancer patients and survivors in Chinese communities. One of the botanical drugs often prescribed by Chinese medicine practitioners to CRC patients in mainland China and in Hong Kong SAR, Baijiangcao, was selected for evaluating its potential influence on FOLFOX treatment in colon cancer mouse models in the present study. A meta-analysis showed that Baijiangcao is one of the commonly used herbal medicines combined with 5-FU-based chemotherapy for CRC treatment (Chen et al., 2019). According to Chinese classical pharmacological literature, the aerial parts of plant species *P. scabiosaefolia* Link [Caprifoliaceae] and *Patrinia villosa* (Thunb.) Dufr. [Caprifoliaceae] are generally regarded as Patriniae Herba (Baijiangcao) (He et al., 2017). Previous study reported that the ethanolic extract of *Patrinia scabiosaefolia* could inhibit 5-FU-resistant colorectal carcinoma cells (Huang et al., 2019). In view of the popularity and availability nowadays, *P. villosa* (PV) is more commonly sold as Baijiangcao than the other allied species such as *P. scabiosaefolia* (Chen et al., 2020) in the herbal markets in central and Southern parts of China and is often prescribed to CRC patients and survivors. Thus, PV, in which over two hundred known metabolites, mainly flavonoids, organic acids, and iridoids have been identified (He et al., 2017; Gong et al., 2021), was used in the present study. The anti-tumor effects of saponins of PV have been reported in cervical cancer and colon cancer mouse models (Zhang et al., 2008; Xia et al., 2018). PV ethanol extract also exhibited therapeutic effect in azoxymethane/dextran sodium sulfate-induced colon cancer mice (Wang et al., 2022; Li et al., 2023). However, the traditional way of preparing Chinese herbal medicines is boiled in water and patients consume the decoction. Therefore, our previous study has also revealed for the first time that PV hot water extract exerted anti-tumor and anti-metastatic effects in colon cancer cells and tumor-bearing mouse models via modulation of TGF-β R1-smad2/3-E-cadherin and FAK-RhoA-cofilin pathways (Yang et al., 2023).

As a matter of fact, since the herbal extracts may be used by CRC patients who are undergoing FOLFOX chemotherapy, it is essential to determine whether herbal extracts would interfere the metabolism of component drugs of FOLFOX. Here, the influence of PV water extract treatment on the levels of dihydropyrimidine dehydrogenase (for 5-FU), serine hydroxymethyltransferase (for folinic acid) and cysteine, methionine and reduced glutathione (for oxaliplatin), in colon tumor-bearing mice have been evaluated. The possible synergy between FOLFOX and PV water extract treatment in the aspects of anti-tumor and anti-metastatic effects in human colon xenograft-bearing mouse as well as syngeneic colon tumor-bearing mouse models were also investigated.

## 2 Materials and methods

### 2.1 Materials

#### 2.1.1 Chemotherapeutics

The 5-fluorouracil in powder form was purchased from Sigma-Aldrich (MA, United States), while its liquid injection form was purchased from Ebewe Pharma (Austria). Folinic acid and oxaliplatin were purchased from Wako Chemicals (Tokyo, Japan).

#### 2.1.2 Herbal materials

**FIGURE 1:**
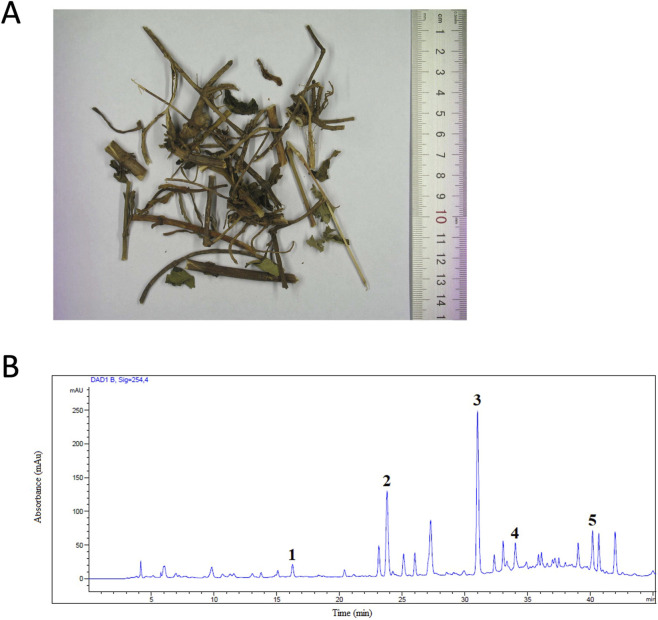
**(A)** Dried herb of *Patrinia villosa* (PV). **(B)** HPLC profile (254 nm) of PV water extract. Chemical markers: 1: gallic acid, 2: protocatechuic acid, 3: chlorogenic acid, 4: caffeic acid, and 5: isochlorogenic acid A.

A single batch of dried herb of *P. villosa* (PV) was purchased from a renowned supplier in Hong Kong and was stored in a temperature- and humidity-controlled store room. The herbal materials (Figure 1A) were authenticated using the comprehensive morphological, chemical and molecular authentication methodology established in our laboratory (Wong et al., 2024). Authenticated PV voucher specimen (No. 3705) was deposited in museum of Institute of Chinese Medicine, The Chinese University of Hong Kong. Hot water extract of PV was prepared and lyophilized into powder form for experiments. This extraction method exactly recapitulated the traditional preparation method as PV herbal materials were extracted with hot water, without using any organic solvent. Dried herb of PV (500 g) was soaked in distilled water (5 L) for 1 h and then boiled to 100 °C under reflux for two times. After extraction, the pooled water extract was filtered with graze to remove the unsolvable particles. The filtered supernatant was collected and was evaporated under reduced pressure to dryness. The yield percentage is 20.8% (w/w).

Standardization of the herbal extract was performed in which the chemical profile was registered and representative chemical markers were determined quantitatively using HPLC as previously described (Yang et al., 2023). In brief, 50 mg of PV water extract powder was added into 1 mL of 70% methanol in water and then ultrasonicated for 30 min at room temperature. The mixture was centrifuged for 15 min at 14,000 rpm. The clear supernatant (3 mL) was injected into an Agilent 1,100 series HPLC System equipped with a DAD detector (Agilent Technologies, CA, United States). A Grace™ Alltech™ Alltima™ C18 column (250 mm × 4.6 mm i. d., 5 μm particle size) with gradient elution at a flow rate of 0.8 mL/min was used to perform the chromatographic separations. Mobile phase of the LC system was: (A) Milli-Q water with 0.1% formic acid, and (B) acetonitrile with 0.1% formic acid; gradient: 0–4 min, 2% B; 4–25 min, 2%–15% B; 25–35 min, 15%–30% B; 35–40 min, 30% B; 40–48 min, 30%–98% B; 48–55 min, 98% B. Spectra were recorded from 190 to 400 nm. The contents of the five identified chemical markers, namely, gallic acid, protocatechuic acid, chlorogenic acid, caffeic acid, and isochlorogenic acid A, were quantified in PV water extract as shown in Table 1. Gallic acid, chlorogenic acid and caffeic acid for chemical quantification were purchased from Sigma-Aldrich (MA, United States), while protocatechuic acid and isochlorogenic acid A were from MedChemExpress (NJ, United States). The HPLC profiles of PV water extract with identified chemical markers were shown in Figure 1B.

**TABLE 1:** The retention times and the contents of the five identified chemicals in *Patrinia villosa* water extract.

Chemicals	Retention times (min)	Contents (±S.D.)^a^(mg/g) (n = 3)
Gallic acid	16.23	0.680 ± 0.001
Protocatechuic acid	23.77	2.580 ± 0.009
Chlorogenic acid	30.99	11.795 ± 0.043
Caffeic acid	34.01	1.009 ± 0.002
Isochlorogenic acid A	40.17	1.998 ± 0.007

^a^ S.D., refers to standard deviation.

#### 2.1.3 Animals and cell lines

Nude mice and BALB/c mice were provided by Laboratory Animal Services Center, The Chinese University of Hong Kong. The experiment protocol was approved by the Animal Experimentation Ethics Committee of The Chinese University of Hong Kong (Ref. No. 21-259-HMF). Human colon cancer cell lines HCT-116, HT-29, SW480 and mouse colon cancer cell line Colon-26 were purchased from American Type Culture Collection (VA, United States). HCT-116 and HT-29 cells were cultured in McCoy’s 5A medium, whereas SW480 and Colon-26 cells were maintained in DMEM or RPMI-1640 medium, respectively. Culture media were supplemented with 10% (v/v) fetal bovine serum and 1% penicillin/streptomycin. These cell lines were cultured at 37 °C in a humidified atmosphere containing 5% CO_2_. All culture media and supplements were purchased from Thermo Fisher Scientific (MA, United States).

### 2.2 Human colon xenograft-bearing mouse model

**FIGURE 2:**
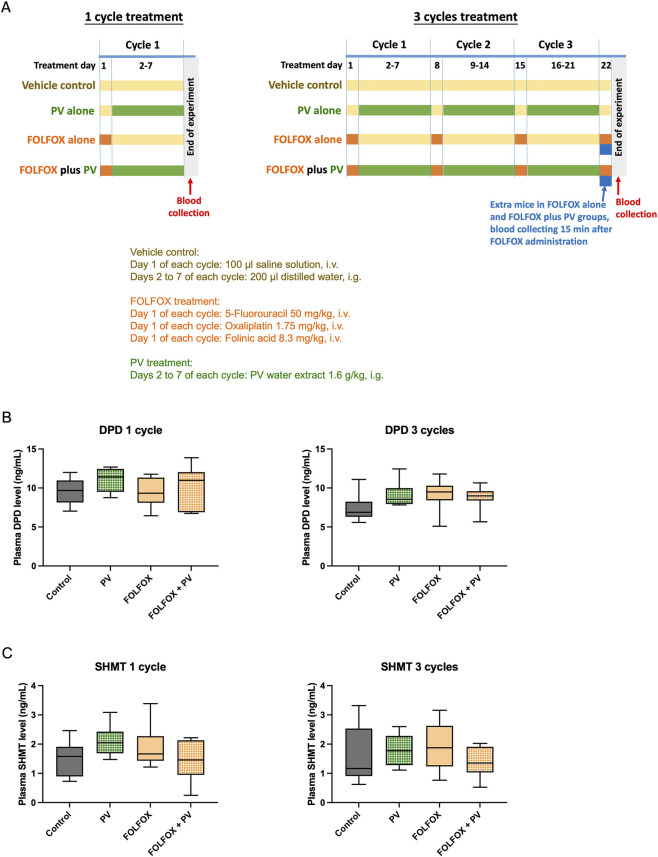
**(A)** Diagram showing the treatments protocol and the time of blood collection during experiments. Levels of **(B)** dihydropyrimidine dehydrogenase (DPD) and **(C)** serine hydroxymethyltransferase (SHMT) in plasma of HCT-116 xenograft-bearing mice receiving different treatments for 1 cycle or 3 cycles. 1-cycle treatment: Control group (n = 8), PV treatment group (n = 9), FOLFOX treatment group (n = 9), FOLFOX + PV treatment group (n = 9); 3-cycle treatment: Control group (n = 8), PV treatment group (n = 9), FOLFOX treatment group (n = 9), FOLFOX + PV treatment group (n = 11). Data were presented in box plots. No significant difference was detected among groups (One-way ANOVA followed by Tukey’s multiple comparison test).

HCT-116 cells (5 × 10^6^ cells) were injected subcutaneously in the flank of nude mice (6–8 weeks of age). After 8–10 days, the size of palpable xenografts was measured using a calliper. The xenograft-bearing mice were randomized into different groups, and were then treated with FOLFOX and/or PV water extract (dosages described in Section 2.4) for 22 days (treatment protocol shown in Figure 2A). Three batches of experiments have been conducted, with the initial number of mice per group were 15. During treatment period, some mice reached the humane endpoint and were euthanized before the end of experiments. Hence, the final mouse numbers in each group: control group (n = 11), PV treatment group (n = 13), FOLFOX treatment group (n = 15), FOLFOX + PV treatment group (n = 13). The body weight and size of xenografts were measured twice weekly. At the end of treatment period, mice were anaesthetized and blood was collected by intra-cardiac puncture. Plasma was obtained by centrifugation and stored at −80 °C until analysis for several key enzymes/molecules involved in the metabolism of FOLFOX component drugs. The tumors were excised from the mice after cervical dislocation for histological analysis.

### 2.3 Syngeneic orthotopic colon tumor-bearing mouse model

The effects of FOLFOX and PV water extract were evaluated in syngeneic orthotopic colon tumor-bearing mouse model, in which the changes in tumor microenvironment after drug treatment could be elucidated (Li et al., 2018). Mouse Colon-26 tumor cells (5 × 10^4^ cells per 50 μL PBS) were injected into the posterior wall of the rectum of BALB/c mice under anaesthesia. Three days after cell inoculation, mice were randomized into different groups and started to receive treatments (same as in the HCT-116 xenograft-bearing mice) for 22 days. Three batches of experiments have been conducted, with the initial number of mice per group were 15. During treatment period, some mice reached the humane endpoint and were euthanized before the end of experiments. Hence, the final mouse numbers in each group: control group (n = 11), PV treatment group (n = 12), FOLFOX treatment group (n = 13), FOLFOX + PV treatment group (n = 15). At the end of treatment period, the mice were anaesthetized and blood was collected in heparinized tube by intra-cardiac puncture. Plasma was obtained by centrifugation and stored at −80 °C until determination of plasma enzyme levels [aspartate transaminase (AST) and alanine transaminase (ALT) for liver function, creatine kinase (CK) for tissue damage] using enzyme kits according to the procedures from manufacturer (Stanbio Laboratory, TX, United States). The tumors, lungs and livers were excised from the mice after cervical dislocation for further histological analysis. The cell proliferation (Ki67 staining), apoptosis (TUNEL staining), and angiogenesis (CD31 staining) in tumor sections were assessed using immunohistochemical analysis as previously described (Li et al., 2017). The livers and lungs were subjected to histological examination for metastasis level assessment. Besides, the population changes of intra-tumoral macrophages and myeloid-derived suppressor cells (MDSC), as well as regulatory T cells (Treg) isolated from lymph nodes were determined by flow cytometry as described in our previous study (Yang et al., 2023).

### 2.4 Treatment with FOLFOX and/or PV water extract

FOLFOX is widely administered through continuous infusion to treat CRC patients. Therefore, the route of FOLFOX administration remained intravenous in the present animal study, while PV water extract was administered to mice via gavage feeding to mimic the situation of cancer patients taking Chinese medicine decoction orally. FOLFOX, consist of 5-fluorouracil (50 mg/kg), oxaliplatin (1.75 mg/kg), folinic acid (8.3 mg/kg), was administered intravenously on day 1 every week, while PV (1.6 g/kg) was given by oral gavage on days 2–7 of each week. The treatment lasted for 22 days.

### 2.5 Measurement of metabolizing enzymes/molecules of FOLFOX component drugs

Blood and liver samples collected from HCT-116 xenograft-bearing mice were used for determination of dihydropyrimidine dehydrogenase (DPD), serine hydroxymethyltransferase (SHMT) and cysteine/methionine/reduced glutathione, which are responsible for the catabolism or transformation of 5-fluorouracil, folinic acid and oxaliplatin, respectively. DPD and SHMT levels were determined using ELISA kits (MyBioSource, CA, United States), and cysteine/methionine/reduced glutathione levels were measured using assay kits (Sigma-Aldrich, MO, United States or Cell Biolabs, CA, United States).

During the 3-week treatment with FOLFOX with or without PV water extract, one batch of blood and liver samples (which labelled as 1-cycle) were collected from xenograft-bearing mice received 1-week treatment. Control group (n = 8), PV treatment group (n = 9), FOLFOX treatment group (n = 9), FOLFOX + PV treatment group (n = 9). Another batch of xenograft-bearing mice, which received 3-week treatment, were terminated for blood and liver collection (labelled as 3-cycle). Control group (n = 8), PV treatment group (n = 9), FOLFOX treatment group (n = 9), FOLFOX + PV treatment group (n = 11). Both batches of samples were subjected to determination of metabolizing enzymes/molecules of FOLFOX component drugs using the assay kits according to the manufacturers’ instructions.

### 2.6 Measurement of 5-FU and folinic acid in plasma and tumor using LC-MS

Furthermore, in order to monitor the steady state plasma levels of FOLFOX component drugs in HCT-116 xenograft-bearing mice, at the end of FOLFOX and/or PV treatment, a batch of mice received intravenous injection of FOLFOX, blood and tumor tissues were collected 15 min after the FOLFOX administration. This selected time point was based on the half-life of 5-FU, with mean half-life of elimination from plasma around 13 min. The levels of FOLFOX component drugs in plasma and tumor of mice treated with FOLFOX alone (n = 7) and PV water extract plus FOLFOX (n = 6) were measured.

#### 2.6.1 Reagents

LC-MS analyses were performed with LC-MS grade reagents including acetonitrile and methanol from RCI Labscan Limited (Bangkok, Thailand), and formic acid from Fisher Chemicals (PA, United States). Ultrapure water (18.2 MΩ cm at 25 °C) was obtained from a Millipore® Milli-Q® Academic® ultrapure water system (MA, United States).

#### 2.6.2 Plasma sample preparation

For 20–50 µL of the plasma sample, a 4 × volume of MeOH was added. After vortex-mixing for a minute, the samples were centrifuged at 14,000 rpm for 5 min. The supernatant was then loaded onto Oasis PRiME HLB cartridges (1 cc, 30 mg) (Waters Corporation, MA, United States), allowed to pass through, and collected in 1.7-mL Eppendorf tubes. To the protein precipitate, an addition of 200 µL of 80% MeOH (v/v) was added. After ultrasonication for 3 min and centrifugation at 14,000 rpm for 5 min, the supernatant was passed through the SPE cartridges and collected, after which the cartridges were rinsed with 100 µL of 80% MeOH (v/v) and collected. The SPE eluate was then centrifuged for 10 min at 14,000 rpm. The supernatant was then concentrated using a Gyrozen HyperVAC-LITE vacuum concentrator (Kyonggi-do, South Korea) operating at 50 °C and 1900 rpm for an hour. The dried extracts were redissolved in 200–500 µL of 10% MeOH (v/v) and filtered through 0.22 µm Acrodisc® syringe filters with wwPTFE membrane (Pall Corporation, NY, United States), prior to analysis by LC-MS.

#### 2.6.3 Tumor tissue sample preparation

Tumor tissue samples (20 mg) were accurately weighed in 2.0-mL Eppendorf tubes and mixed with 500 µL of MeOH. After homogenization using a QIAGEN TissueRuptor (Hilden, Germany), an additional volume of MeOH was added to reach a sample-to-solvent ratio of 1:50. After vortex-mixing for 1 min followed by centrifugation at 14,000 rpm at 4 °C for 20 min for protein removal, 800 µL of the supernatant were dried using a vacuum concentrator operating at 50 °C and 1900 rpm for an hour. The dried extracts were redissolved in 160 µL of 10% MeOH (v/v) and filtered through 0.22 µm Acrodisc® syringe filters with PTFE membrane prior to analysis by LC-MS.

#### 2.6.4 Instrumentation

Chromatographic separations were carried out on a Waters ACQUITY UPLC I-Class PLUS System (Waters Corporation) using an Agilent InfinityLab Poroshell 120 Aq-C18 column (3.0 × 100 mm, 2.7 µm) with a matching guard column (3.0 mm × 5 mm, 2.7 µm) (Agilent Technologies, CA, United States) by gradient elution at a flow rate of 0.5 mL/min.

The UPLC system was operating at 35 °C as follows: mobile phase (A) milli-Q water with 0.1% formic acid, and (B) acetonitrile with 0.1% formic acid; gradient: 0–2 min, 1% B; 2–5 min, 1%–30% B; 5–6 min, 30%–98% B; 6–7.5 min, 98% B; 7.5–8 min, 98%–1% B; 8–10 min, 1% B. The temperature of the auto-sampler was maintained at 18 °C. The injection volume was 0.5 μL.

MS analysis was performed using a Waters SQ Detector 2 with Electrospray Ionization Source operating in negative ion mode. Optimization of the MS parameters was performed by direct infusion of standard solutions of 5-FU and folinic acid into the MS. The MS sampling cone voltages for the metabolties were optimized automatically with the use of Waters IntelliStart™ Software. The MS was operated with the following conditions: source temperature, 150 °C; desolvation gas temperature, 500 °C; desolvation gas flow, 1000 L/h; capillary voltage, −2.5 kV; sampling cone voltage, −34 and −54 V for 5-FU and folinic acid, respectively. Selected ion monitoring mode was employed for the acquisition of deprotonated pseudo-molecular ions [M-H^-^] with mass-to-charge (m/z) ratios of 128.92 and 472.16 for the analysis of 5-FU and folinic acid, respectively. The dwell time was 0.128 s. Mass data were collected from 1.5 to 6 min. Waters MassLynx™ software (version 4.2) was used for instrument control and data acquisition, while TargetLynx™ software was used for quantitative analysis (Waters Corporation).

### 2.7 Measurement of platinum content in plasma and tumors

Plasma samples (400 µL) and tumor tissue lysates samples (400 µL) of each mouse treated with FOLFOX and/or PV water extract were sent to Chemical Testing Services, Hong Kong Baptist University, for determination of platinum content. Samples were undergone acid digestion and then measured for platinum content using inductively coupled plasma - mass spectrometry (ICP-MS).

### 2.8 Histological and immunohistochemistry staining

Tumors, lungs and livers excised from mice were fixed in 10% formalin and processed for paraffin embedding. The paraffin embedded lung and liver samples were sectioned at 5 μm and further stained with hematoxylin & eosin (H&E). The tumors sections were subjected to immunohistochemistry staining of Ki67 for cell proliferation, CD31 for angiogenesis and TUNEL staining for apoptosis as described previously (Jiang et al., 2022).

### 2.9 Real-time quantitative PCR (RT-qPCR) of tumor tisssues

Tumor tissues collected from HCT-116 xenograft-bearing mice were extracted for total RNA TRIzol reagent (Thermo Fisher Scientific, MA, United States). RNA (1 μg) was reverse transcribed using EasyScript® One-Step SuperMix (TransGen, Beijing, China). Quantitative real-time PCR (qRT-PCR) was performed with the SYBR Green Master Mix for qPCR (Thermo Fisher Scientific) on a LightCycler® 480 Instrument II (Applied Roche, CA, United States). The primers were synthesized by Integrated DNA Technologies (Singapore) and Table 2 showed the sequences. The mRNA levels of target genes were normalized with to those of GAPDH.

**TABLE 2:** Primer sequences of target genes tested in HCT-116 xenograft tissues.

AKT1-forward	5′-TCT​ATG​GCG​CTG​AGA​TTG​TG-3′
AKT1-reverse	5′-CTT​AAT​GTG​CCC​GTC​CTT​GT-3′
Cofilin-forward	5′-ATA​AGG​ACT​GCC​GCT​ATG​CC-3′
Cofilin-reverse	5′-CTC​TTA​AGG​GGC​GCA​GAC​TC-3′
EGFR-forward	5′-TCC​CTC​AGC​CAC​CCA​TAT​GTA​C-3′
EGFR-reverse	5′-GTC​TCG​GGC​CAT​TTT​GGA​GAA​TTC-3′
FAK-forward	5′-AAT​ACG​GCG​ATC​ATA​CTG-3′
FAK-reverse	5′-CAT​GCC​TTG​CTT​TTC​GCT​GT-3′
GAPDH forward	5′-CGA​GAT​CCC​TCC​AAA​ATC​AA-3′
GAPDH reverse	5′-GGT​GCT​AAG​CAG​TTG​GTG​GT-3′
PIK3CA-forward	5′-TGG​ATG​CTC​TAC​AGG​GCT​TT-3′
PIK3CA-reverse	5′-GTC​TGG​GTT​CTC​CCA​ATT​CA-3′

### 2.10 Statistical analysis

Data in all animal experiments were expressed as mean +S.E.M. Differences among treatment and control groups were analyzed using one-way analysis of variance (ANOVA) followed by *post hoc* tests, depending on data distribution. In all comparisons, value of *p* < 0.05 were considered as statistical significance. All statistical analyses were performed using GraphPad Prism (GraphPad Software, CA, United States).

## 3 Results

### 3.1 Effect of PV water extract treatment on the plasma levels of metabolizing enzymes of 5-FU and folinic acid in human colon xenograft-bearing mice

To determine if PV water extract treatment affect the metabolism of 5-FU and folinic acid, plasma levels of DPD (for 5-FU) and SHMT (for folinic acid) in HCT-116 xenograft-bearing mice were measured. As shown in Figure 2B, oral administration of PV water extract for 1 or 3 cycle(s) did not alter the plasma levels of DPD. Therefore, it would not have any adverse effects on drug metabolism of 5-FU.

The conversion of folinic acid into its active form, 5,10-MTHF, is catalyzed by the vitamin B6-dependent SHMT, with the simultaneous conversion of serine into glycine. As shown in Figure 2C, oral administration of PV water extract or intravenous administration of FOLFOX for 1 or 3 cycle(s) slightly increased the SHMT level, without significant difference among the treatment groups. In contrast, combine treatment of PV water extract and FOLFOX seems to reverse the increases of plasma SHMT level, but its effect was insignificant when compared to that in FOLFOX alone group.

### 3.2 Effect of PV water extract treatment on the plasma levels of molecules affecting oxaliplatin conversion in human colon xenograft-bearing mice

**FIGURE 3:**
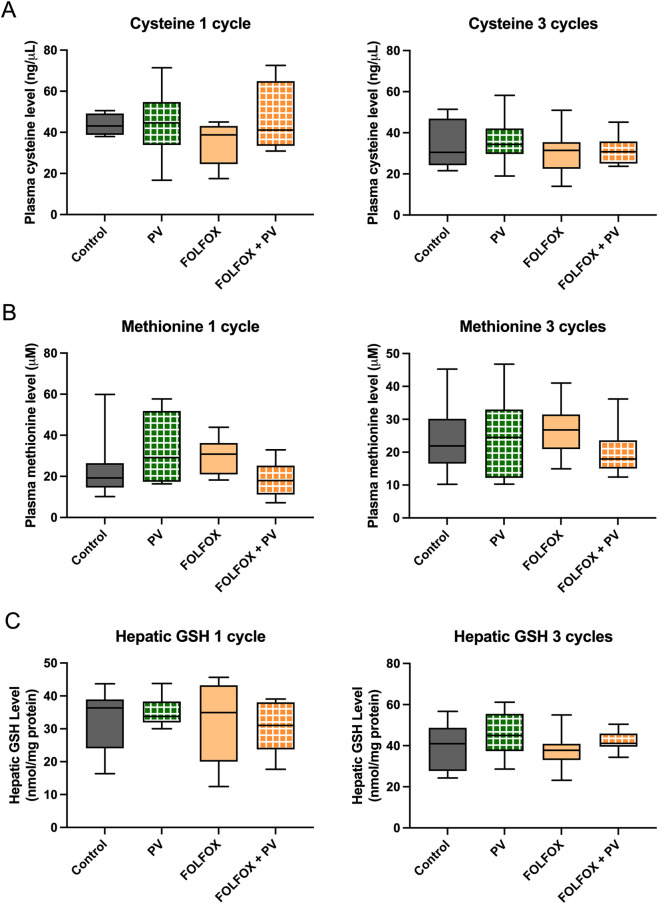
Levels of **(A)** plasma cysteine, **(B)** plasma methionine and **(C)** hepatic reduced glutathione (GSH) in HCT-116 xenograft-bearing mice receiving different treatments for 1 cycle or 3 cycles. Data were presented in box plots [1-cycle treatment: Control group (n = 8), PV treatment group (n = 9), FOLFOX treatment group (n = 9), FOLFOX + PV treatment group (n = 9); 3-cycle treatment: Control group (n = 8), PV treatment group (n = 9), FOLFOX treatment group (n = 9), FOLFOX + PV treatment group (n = 11)]. No significant difference was detected among groups (One-way ANOVA followed by Tukey’s multiple comparison test).

Oxaliplatin undergoes rapid and non-enzymatic biotransformation to form monochloro-, dichloro- or diaquo-DACH that interferes with DNA synthesis. However, in the presence of cysteine, methionine or reduced glutathione (GSH), oxaliplatin is converted into chemically unreactive species. Plasma levels of these molecules in HCT-116 xenograft-bearing mice were determined. As shown in Figure 2, cysteine (Figure 3A) and methionine (Figure 3B), as well as hepatic GSH (Figure 3C) levels were not significantly affected by PV water extract intervention. There was no statistical difference between FOLFOX and FOLFOX plus PV groups. It implies that PV water extract would neither increase nor decrease the inactive forms of oxaliplatin.

Therefore, taken altogether, oral administration of PV water extract at the tested dose would not affect drug metabolism of FOLFOX in HCT-116 xenograft-bearing mice.

### 3.3 Effect of PV water extract treatment on the concentrations of FOLFOX component drugs in plasma and tumors

**FIGURE 4:**
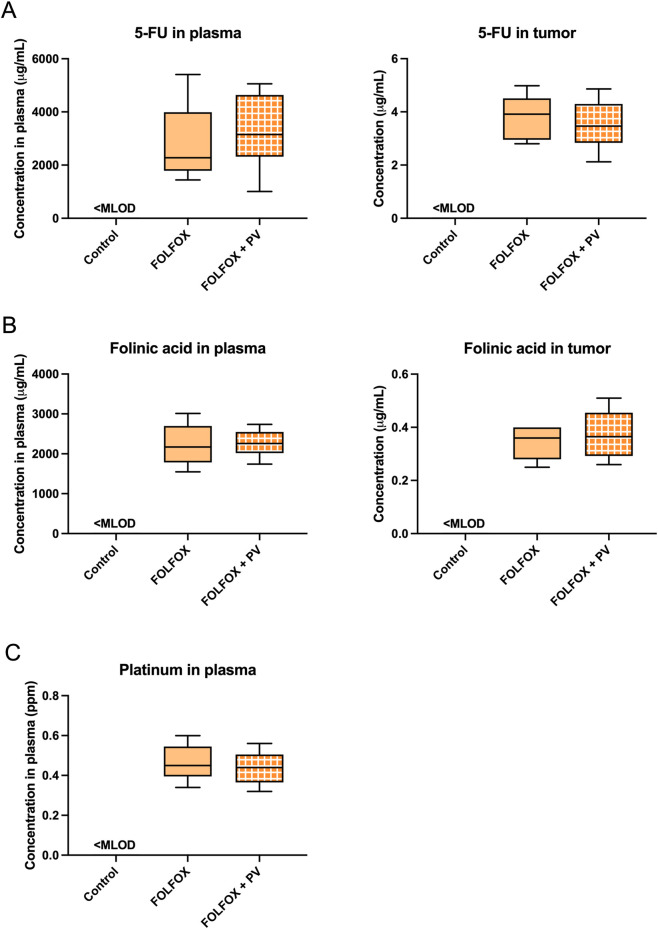
Levels of FOLFOX component drugs **(A)** 5-fluorouracil (5-FU), **(B)** folinic acid, **(C)** platinum in plasma or tumor tissue lysate of HCT-116 xenograft-bearing mice. Blood was collected 15 min after FOLFOX treatment on day 23 (the end of PV water extract treatment). Data were presented in box plots, n = 6–7. No significant difference was detected among groups (One-way ANOVA followed by Tukey’s multiple comparison test). MLOD refers to method detection limit. The MLODs of 5-FU, folinic acid and platinum were 0.048 μg/mL, 0.013 μg/mL and 0.02 ppm of plasma samples, respectively. The MLODs of 5-FU, folinic acid and platinum were 0.11 μg/g, 0.13 μg/g and 0.01 ppm of tumor tissue samples, respectively.

To further monitor the steady state plasma levels of FOLFOX component drugs in HCT-116 xenograft-bearing mice, at the end of treatment (day 23), mice of both FOLFOX alone group and FOLFOX plus PV group were intravenously administered with FOLFOX. Blood and tumor tissues were collected 15 min after the FOLFOX administration. The plasma levels of FOLFOX component drugs were determined using HPLC (5-FU and folinic acid) and ICP-MS (oxaliplatin in the form of platinum). Results showed that there is no significant difference of 5-FU, folinic acid and platinum levels in plasma of mice treated with or without PV water extract (Figures 4A–C). Furthermore, the levels of 5-FU and folinic acid in tumor tissue lysate were not different in FOLFOX alone group and FOLFOX plus PV group. However, the level of platinum in tumor tissue lysate was under detection limit (0.01 ppm). These data suggested that the presence of PV water extract did not affect the concentrations of FOLFOX component drugs in colon xenograft-bearing mice, indicating that the amount of FOLFOX component drugs would not be affected by oral administration of PV water extract for 3 weeks.

### 3.4 Efficacy of FOLFOX and/or PV water extract treatments in human colon xenograft-bearing mice

**FIGURE 5:**
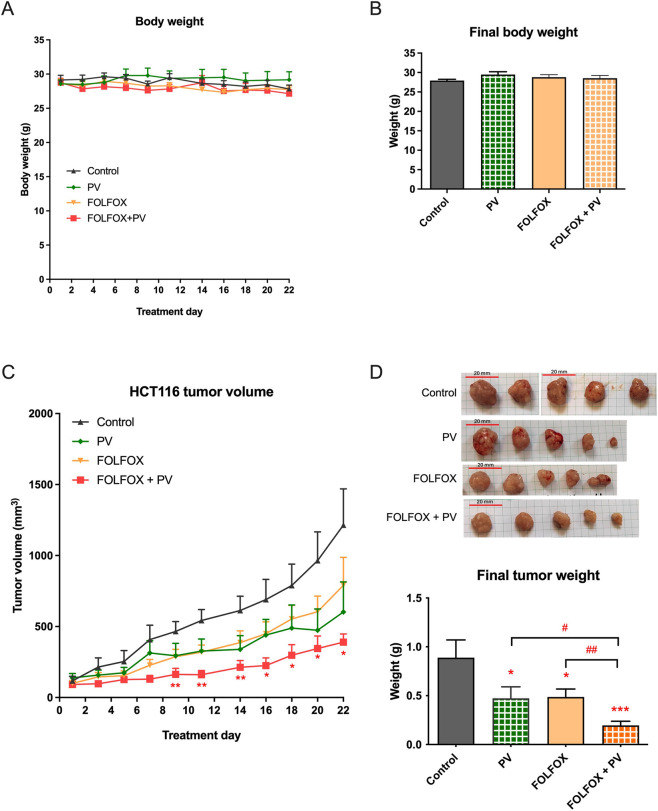
Effect of FOLFOX and/or *Patrinia villosa* (PV) water extract treatments on the growth of tumors in HCT-116 xenograft-bearing mice. **(A)** Body weight of mice during treatments; **(B)** final body weight of mice; **(C)** tumor volume during the treatments; **(D)** representative photos of tumors in each treatment groups and bar chart showing final tumor weight. All data were expressed as mean +S.E.M. (n = 11–15). *p < 0.05, **p < 0.01, ***p < 0.001 as compared to the control group (One-way ANOVA followed by Tukey’s multiple comparison test). ^#^ *p* < 0.05, ^##^ *p* < 0.01 as compared between groups. Control group (n = 11), PV treatment group (n = 13), FOLFOX treatment group (n = 15), FOLFOX + PV treatment group (n = 13).

Apart from the metabolism of FOLFOX, the potential influence by PV water extract on efficacy of FOLFOX has also been evaluated. The anti-proliferative responses of human colon cancer cells (HCT-116, HT-29, SW480) towards PV water extract were determined and compared using MTT assay. The IC_50_ values of PV water extract in HCT-116, HT-29 and SW480 cell lines were 393.0 ± 34.8 μg/mL, 751.1 ± 27.5 μg/mL, 689.7 ± 23.9 μg/mL, respectively. HCT-116 was shown to be the most sensitive cell line with the lowest IC_50_ value among all tested cell lines. Thus, HCT-116 cells were used to establish xenografts in nude mice, which received FOLFOX and/or PV water extract treatments. Results showed that the 3-week treatments did not cause significant change at body weight of mice in all groups as shown in Figures 5A,B. The tumor volume in mice treated with FOLFOX plus PV was significantly decreased from Day 9 onwards (*p* < 0.05), while the FOLFOX and PV alone exhibited mild inhibitory effect on tumor growth (Figure 5C). After the 3-week treatment, mice were euthanized and tumors were collected. The final tumor weights in all treatment groups were found to be significantly reduced when compared to untreated control (*p* < 0.05, Figure 5D), indicating the inhibitory effects of PV and/or FOLFOX on HCT-116 xenograft growth. Notably, the final tumor weight of FOLFOX plus PV group was the lowest, and was significantly lower than those of FOLFOX or PV alone treatment (*p* < 0.05), suggesting that it was the most effective regimen.

**FIGURE 6:**
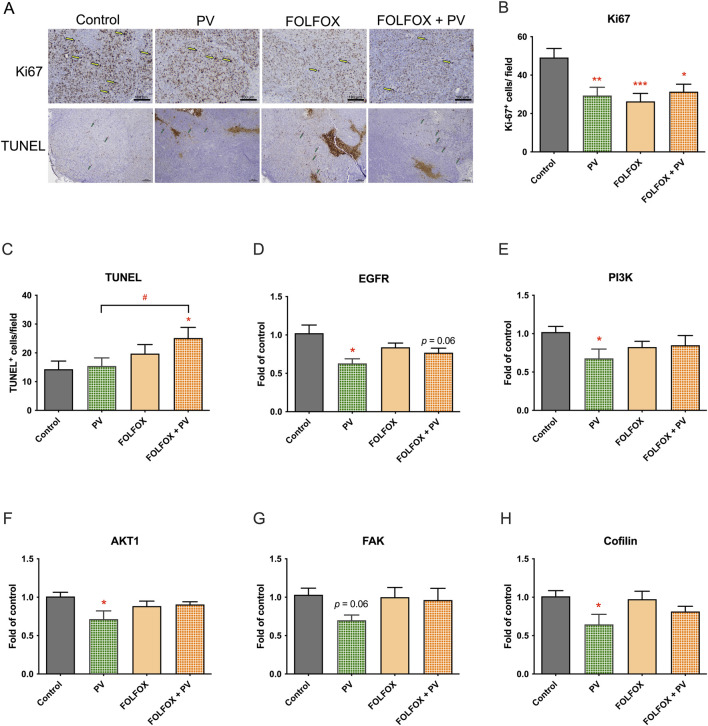
Effect of FOLFOX and/or *Patrinia villosa* (PV) water extract treatments on tumor growth in HCT-116 xenograft-bearing mice. **(A)** Representative images of tumor sections stained with Ki67 and TUNEL. Yellow and green arrows indicated positive-stained cells in the tumor sections, scale bar = 100 µm. Quantitative results of **(B)** Ki67 and **(C)** TUNEL positive-stained cells in tumors of different treatment groups. Data were presented as mean +SEM, n = 11–15. **(D–H)** Quantitative RT-PCR of *EGFR, PIK3CA, AKT1, FAK* and *cofilin* mRNA expressions in HCT-116 xenografts were performed. Data were normalized to GAPDH expression in xenografts of control group. mRNA expression was shown as fold of control. Differences between treatment and control groups was analyzed using one-way ANOVA followed by Tukey’s multiple comparison test. *p < 0.05, **p < 0.01, ***p < 0.001 vs. control group. ^#^ *p* < 0.05 as compared between groups. Control group (n = 11), PV treatment group (n = 13), FOLFOX treatment group (n = 15), FOLFOX + PV treatment group (n = 13).

Furthermore, the cell proliferation and apoptosis level in tumor sections from mice of all groups were assessed by immunohistochemical staining. As shown in Figures 6A,B, the number of Ki67 positive-stained cells (proliferative cells) was significantly reduced in the PV alone, FOLFOX alone and FOLFOX plus PV groups (*p* < 0.05), whereas the number of TUNEL positive-stained cells (apoptotic cells) was significantly increased in FOLFOX plus PV group only when compared to control group (Figures 6A,C). Although there was no statistical difference among PV alone, FOLFOX alone and the combined treatment groups, the more potent anti-tumor activity of combined treatment could be observed.

Since our previous studies reported that water extract of Herba Patriniae (including PV) could downregulate the expression of EGFR, PI3K, AKT, FAK and cofilin in HCT-116 cells (Yang et al., 2021), the mRNA expressions of these genes in HCT-116 xenografts were determined using qPCR. Results showed that HCT-116 xenografts from mice treated with PV alone have lower expression of *EGFR, PIK3CA, AKT1* and *Cofilin*, with significant difference from those from untreated control mice (*p* < 0.05, Figures 6D–F,H). FOLFOX alone treatment slightly suppressed the mRNA expression of *EGFR* and *P13K* and *AKT1*. While the presence of PV in the combined treatment did not significantly change of the expression of these genes.

### 3.5 Efficacy of FOLFOX and/or PV water extract treatments in syngeneic orthotopic colon tumor-bearing mice

**FIGURE 7:**
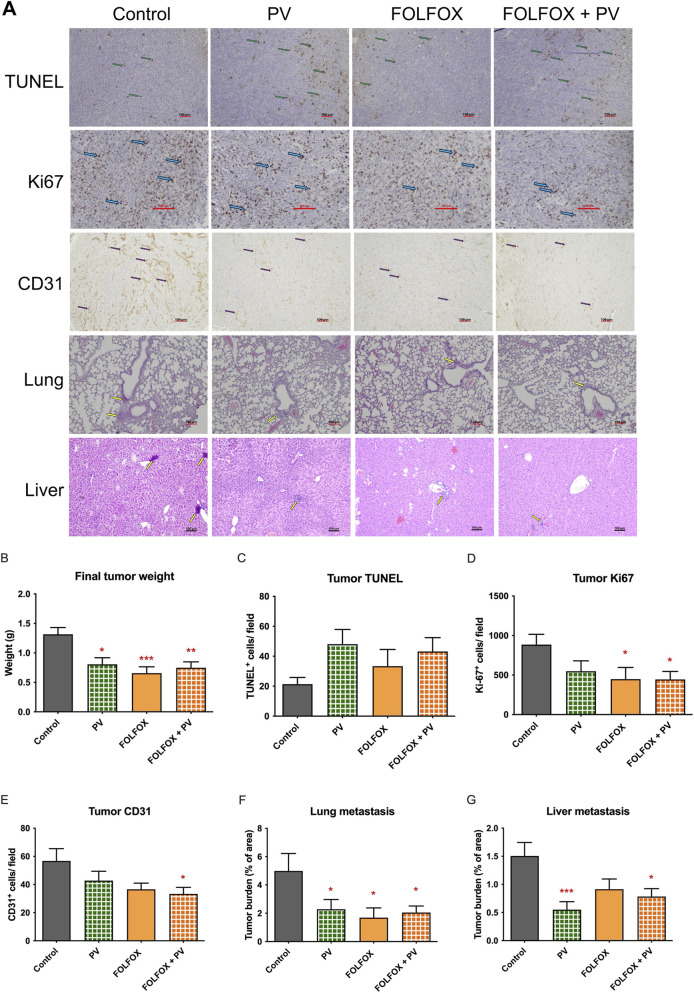
Effects of FOLFOX and/or *Patrinia villosa* (PV) water extract on tumor growth and lung and liver metastasis in Colon-26 tumor-bearing mice. **(A)** Representative images of tumor sections stained with Ki67, TUNEL and CD31 as well as lung and liver sections stained with H&E. Green, light blue and purple arrows indicated positive-stained cells in the tumor sections. Yellow arrows showed the tumor nodules in lung or liver section, scale bar = 100 µm. **(B)** Final weight of Colon-26 tumors, **(C)** TUNEL, **(D)** Ki67 and **(E)** CD31 positive-stained cells in tumors of mice of different treatment groups were recorded. Tumor burden in **(F)** lung and **(G)** liver of mice were assessed. Data were presented as mean +SEM, n = 11–15. Differences between treatment and control groups was analyzed using one-way analysis of variance (ANOVA) followed by Tukey’s multiple comparison test. *p < 0.05, **p < 0.01, ***p < 0.001 as compared to untreated control group. Control group (n = 11), PV treatment group (n = 12), FOLFOX treatment group (n = 13), FOLFOX + PV treatment group (n = 15).

In colon-26 tumor bearing mice, results showed that final tumor weights in all treatment groups were significantly reduced when compared to untreated control (*p* < 0.01, Figure 7B), indicating the inhibitory effects of PV and/or FOLFOX on Colon-26 tumor growth. Besides, the cell proliferation and apoptosis level in tumor sections from PV alone, FOLFOX alone, and FOLFOX plus PV groups were further assessed by immunohistochemical staining. As shown in Figures 7A,C, the number of TUNEL positive-stained cells (apoptotic cells) were increased in PV alone and FOLFOX plus PV group when compared to control group, although the differences were not statistically significant. Nonetheless, the numbers of Ki67 positive-stained cells (proliferative cells, Figure 7D) and CD31 positive-stained cells (endothelial cells, Figure 7E) were significantly reduced in the FOLFOX plus PV groups (*p* < 0.05).

Furthermore, the metastasis levels in lungs and livers of tumor-bearing mice have also been assessed. Results showed that PV alone, FOLFOX alone, and FOLFOX plus PV treatments all significantly reduced the lung metastasis (*p* < 0.05, Figure 7F). However, apparent decrease of liver metastasis was observed in PV alone and the FOLFOX plus PV treatment groups (*p* < 0.05), but not in FOLFOX alone group (Figure 7G).

**FIGURE 8:**
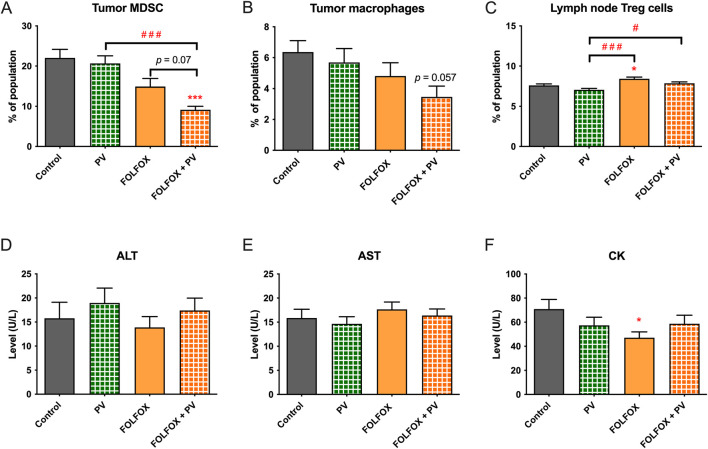
Effects of FOLFOX and/or *Patrinia villosa* (PV) on immune responses in Colon-26 tumor-bearing mice. Populations of **(A)** tumor myeloid-derived suppressor cells (MDSC), **(B)** tumor macrophages, **(C)** regulatory T cells in lymph node were determined using flow cytometry. Levels of **(D)** of alanine transaminase (ALT), **(E)** aspartate transaminase (AST) and **(F)** creatine kinase (CK) in plasma of Colon-26 tumor-bearing mice were determined. Data were presented as mean +SEM, n = 11–15. Differences between treatment and control groups was analyzed using one-way analysis of variance (ANOVA) followed by Tukey’s multiple comparison test. *p < 0.05, ***p < 0.001 as compared to untreated control group. ^#^ *p* < 0.05, ^###^ *p* < 0.001 as compared among groups. Control group (n = 11), PV treatment group (n = 12), FOLFOX treatment group (n = 13), FOLFOX + PV treatment group (n = 15).

For the immunomodulation in tumor-bearing mice, the populations of intra-tumoral macrophages and myeloid-derived suppressor cells (MDSC) as well as regulatory T cells in lymph nodes were determined using flow cytometry. Results showed that the percentage of MDSC in tumors was significantly reduced after FOLFOX plus PV treatment when compared to control and PV alone groups (*p* < 0.001) and was marginally significant when compared with FOLFOX alone group (*p* = 0.07, Figure 8A). While the percentage of tumor macrophages in FOLFOX alone and FOLFOX plus PV groups were lower than those in control and PV alone groups, though no statistically significant difference was obtained (Figure 8B). In contrast, the regulatory T cells (Treg) in lymph nodes of tumor-bearing mice were significantly increased in FOLFOX alone group when compared to control group (*p* < 0.05), whereas the percentage of Treg was lower in PV and FOLFOX plus PV groups when compared with FOLFOX alone group (Figure 8C).

On the other hand, plasma was obtained from blood by centrifugation. The determination of plasma enzyme levels [aspartate transaminase (AST) and alanine transaminase (ALT) for liver function, creatine kinase (CK) for tissue damage were performed. Results showed that there was no statistical difference of the enzyme levels of ALT and AST among Control, PV alone, FOLFOX alone and the FOLFOX plus PV treatment groups (Figures 8D,E). However, FOLFOX alone significantly decreased the plasma level of CK (Figure 8F), while the FOLFOX plus PV treatment reversed such change.

## 4 Discussion

In Chinese communities worldwide, herbal medicines are very often prescribed by Chinese medicine practitioners to cancer patients. However, clinicians and oncologists have great concern on the use of herbal medicines during chemotherapy due to unknown herb-drug interaction. This study attempted to provide an answer for the question: would herbal medicines treatment affect metabolism of the chemotherapeutic drugs? Here, taken CRC treatment as an example, chemotherapeutic regimen FOLFOX was applied in colon cancer mouse models weekly, while PV water extract was administered to mice daily, except the FOLFOX injection day. Then, the plasma levels of selected metabolism enzymes and precursor molecules of component drugs of FOLFOX in mice as well as the anti-tumor efficacy were determined after treatments. Our study revealed for the first time that 3-week herbal extract PV treatment did not show significant interference on the levels of metabolic enzyme of 5-FU and folinic acid as well as the regulatory molecules involved in oxaliplatin conversion. In addition, the beneficial outcomes of combined FOLFOX and PV water extract treatments in colon tumor-bearing mice in terms of inhibiting tumor progression and metastasis, modulating tumor microenvironment were demonstrated.

The treatment protocol in this study (Figure 2A) was designed to mimic the clinical setting of CRC patients undergone chemotherapy with FOLFOX and taken herbal medicine PV water extract in the same period. When designing the dosing intervals between FOLFOX and PV water extract, half-life of FOLFOX drugs have been considered. Among the three component drugs of FOLFOX, the half-life of 5-FU is the shortest, with mean half-life of elimination from plasma only around 13 min, with a range of 5–27 min. Due to rapid metabolic elimination, 5-FU was no longer detectable after 2 h following intravenous injection (Heggie et al., 1987). Besides, the elimination half-life of folinic acid is also short, only ranged from 0.9 to 1.9 h (Greiner et al., 1989). On the other hand, the half-life of oxaliplatin is relatively long. Long terminal half-life for ultrafilterable platinum (9.32–29.17 h) was observed (Cho et al., 2006; Burz et al., 2009). However, by 24 h after administration, platinum is almost entirely conjugated to low molecular weight amino acids which is probably not clinically relevant (Delord et al., 2003). Therefore, it was believed that 24 h will be long enough to space FOLFOX and PV water extract apart during the treatment. In another aspect, the measurement of 5-FU, folinic acid and platinum at a single time point (15 min post-dose) might not be able to assess the overall exposure or clearance of the drugs, which might regard as a limitation of the present protocol.

During the 3-week FOLFOX and/or PV water extract treatments, blood were collected for determination of levels of DHD (for 5-FU), SHMT (for folinic acid), as well as cysteine, methionine and reduced glutathione (for oxaliplatin) after the first cycle of treatment, so as to evaluate if the PV treatment would affect these enzymes/molecules within short period of time. While the blood collected at the end of treatment (3-cycle), the potential influence of PV treatment on the metabolism of FOLFOX component drugs and the efficacy of FOLFOX could be examined in this study. Results showed that the levels of metabolising enzymes/molecules of FOLFOX component drugs were not affected by PV treatment. Although a previous study reported that the metabolites luteolin, which was present in PV extract (Yan et al., 2016), could decrease dihydropyrimidine dehydrogenase (DHD) mRNA level in hepatocarcinoma cells (Xu et al., 2016), the plasma levels of this enzyme have not been affected by PV water extract treatment in our colon tumor-bearing mice. Furthermore, the concentrations of 5-FU, folinic acid and platinum in plasma and tumors after 3-cycle treatments have also been measured and there was again no significant difference between FOLFOX alone and FOLFOX plus PV water extract groups. However, pharmacokinetic parameters of FOLFOX have not been further assessed in the present study which is a limitation of the study. Our data may not entirely substitute for a quantitative evaluation of pharmacokinetic parameters, which directly reflect systemic drug exposure and are critical for assessing herb–drug interaction risk.

There were some limitations of the original experimental design, in which the levels of selected enzymes and precursor molecules of FOLFOX component dugs were measured in PV-treated and untreated mice plasma (and liver lysate for GSH only). It was understood that liver is the main organ responsible for the catabolism of 5-FU and thus the primary site for DPD activity. In a pilot test, we have tried measuring DPD levels in liver homogenate and plasma samples obtained from tumor-bearing mice receiving FOLFOX treatment. However, DPD levels in liver homogenate were too low/unstable to be detected. On the other hand, DPD levels in plasma could be easily measured using the same ELISA kit. For oxaliplatin, its degradation in whole blood plays a key role for the elimination of the drug. Once administered intravenously, oxaliplatin undergoes rapid and non-enzymatic biotransformation to form monochloro-, dichloro- or diaquo-DACH that interferes with DNA synthesis. However, it also readily reacts with endogenous low-molecular-weight species such as cysteine, methionine and reduced glutathione (GSH) to form chemically unreactive species. At the very beginning, we planned to measure plasma levels of cysteine, methionine and GSH. However, GSH levels of some mouse plasma samples were very low [below detection limit of assay kit (0.5 nmol/mL)]. Therefore, hepatic GSH was detected instead. Plasma might not be the most appropriate biological source for all aspects. However, we may interpret the data of enzymes/molecules involved in metabolism of FOLFOX component drugs as supplementary information to metabolite results obtained from LC-MS or ICP-MS. Moreover, for future clinical study, measurement of plasma parameters will be relatively more feasible for continuous herb-drug interaction monitoring in patients receiving PV and FOLFOX regimen.

Apart from the metabolism of component drugs of FOLFOX, the anti-tumor efficacy of the combined FOLFOX and PV water extract treatment would also be crucial to justify the use of such combination. The findings from the human colon HCT-116 xenograft-bearing mice showed that PV water extract treatment enhanced the anti-tumor efficacy of low dose FOLFOX (which is 1/16 of the clinical dose), with increased apoptotic cells in tumors as shown in TUNEL staining. Although a few proliferation-related genes, *EGFR, PIK3CA, AKT1* and *Cofilin*, were downregulated in tumors of PV treatment alone group, such decrease was not observed in FOLFOX nor FOLFOX plus PV groups, it was possible that FOLFOX at low dose could not elicit regulation on these genes or these genes are not the major targets of FOLFOX component drugs. Nevertheless, a few studies reported the potentiation effects of some major metabolites (e.g., four out of five chemical markers identified in our PV water extract, namely, gallic acid, protocatechuic acid, chlorogenic acid, caffeic acid) towards 5-FU or oxaliplatin, the component drugs of FOLFOX. For example, gallic acid was shown to co-crystallize with 5-FU and exhibit synergistic anti-tumor activity in breast cancer *in vivo* (Hao et al., 2023). While in gastric adenocarcinoma cells, protocatechuic acid combined with 5-FU could promote apoptosis via *p53* and *Bcl-2* signaling pathways (Motamedi et al., 2020). Besides, chlorogenic acid was shown to enhance 5-FU induced decrease of proliferation in hepatocellular carcinoma cells, via suppressing ERK activation through the overproduction of ROS (Yan et al., 2015). Recent study also showed that chlorogenic acid reduced intestinal mucositis in mice caused by 5-FU by altering SIRT1 signaling-mediated oxidative stress pathway (Lin et al., 2025). In the presence of low concentration of chlorogenic acid, the activity of oxaliplatin in cervical cancer cells could be enhanced (Catanzaro et al., 2016). Lastly, caffeic acid was shown to enhance apoptosis of HCT-116 colon cancer cells when combined with oxaliplatin (Lim et al., 2024). In fact, PV water extract preparation followed the traditional way of preparing Chinese herbal medicines, that is boiled in water and patients consume the decoction. Taking together, the PV water extract containing the metabolites could exert certain potentiation on the anti-tumor efficacy of FOLFOX, as observed in the colon tumor-bearing mice in this study.

In view of the dosage regimen of FOLFOX for mice, it was calculated from clinical doses (Maindrault-Goebel et al., 2001): 5-fluorouracil (2,400 mg/m^2^), oxaliplatin (85 mg/m^2^), folinic acid (400 mg/m^2^) to mouse theoretical dose 5-fluorouracil (800 mg/kg), oxaliplatin (28 mg/kg), folinic acid (133 mg/kg), according to the conversion table listed in US FDA guidance (Center for Drug Evaluation and Research, 2005). In clinical practice FOLFOX dosing is a 14-day cycle for 12 times in 6 months. However, due to the relatively fast progression of colon tumors in mouse models (generated using Colon 26 cells or MC38 cells), the treatments may not last over 4 weeks (as the tumors will exceed humane endpoint). Thus, FOLFOX is usually administered via intraperitoneal injection once weekly for 3–4 weeks in mouse models (Robinson et al., 2013; Guan et al., 2020). Besides, previous study has suggested the conversion of adult mouse and human age, i.e. 1 mouse day will be equivalent to 30 human days (Wang et al., 2020). Our weekly dosing schedule for 21 days in tumor-bearing mice might have the efficacy of FOLFOX over 600 days in patients. The anti-tumor efficacy of FOLFOX was examined in HCT-116 xenograft-bearing mice in our pilot studies. Results showed that FOLFOX at 1X theoretical dose, 1/2 of theoretical dose, 1/4 of theoretical dose all caused death in mice, while treatment of 1/10 and 1/16 of theoretical doses of FOLFOX could exhibit apparent anti-tumor effects without death. Since the present study aimed to evaluate the combined use of FOLFOX and PV water extract in colon tumor-bearing mouse models, 1/16 of theoretical dose of FOLFOX, i.e., 5-fluorouracil (50 mg/kg), oxaliplatin (1.75 mg/kg), folinic acid (8.3 mg/kg), was chosen for the present combination study. Nevertheless, the use of this low dose FOLFOX (1/16 of theoretical dose) might still deviate from standard preclinical protocols which may be another limitation of the present study.

For the dosage of PV water extract, the clinical dosage of *P. villosa* herb is 9–15 g/person/day according to Chinese Pharmacopoeia (1977 version) and local standards in China (Gong et al., 2021)., the clinical dosage (15 g/person/day) of PV herb was equivalent to 0.4 g/kg in mouse. In our previous study (Yang et al., 2023), 0.4, 0.8 and 1.6 g/kg of PV water extract have been evaluated for the anti-tumor and anti-metastatic efficacies in colon tumor-bearing mice, with 1.6 g/kg the most potent. Therefore, PV (1.6 g/kg) was applied in the present study.

On the other hand, the results from the syngeneic colon tumor-bearing mouse model also demonstrated that PV water extract would not affect the anti-tumor efficacy of FOLFOX in immuno-competent mice, while the anti-metastasis in liver and anti-angiogenic efficacies of the combined PV and FOLFOX treatment were better than those of PV or FOLFOX alone treatments. Interestingly, PV treatment was found to alter the population of tumor MDSC and macrophage as well as lymph node regulatory T cells in FOLFOX plus PV treatment group. These findings implied that PV treatment might further enhance the anti-tumor effect of FOLFOX by modulating the tumor microenvironment, which is worth to be further investigated.

In summary, the present study elucidated the potential influence of herbal extract PV on the metabolism of chemotherapeutic regimen FOLFOX, as well as the anti-tumor efficacy of FOLFOX in human colon xenograft-bearing mice and immuno-competent mice, which would provide more scientific evidences on the combined use of *P. villosa* water extract during FOLFOX treatment in colon cancer.

## 5 Conclusion

The present study revealed for the first time that no observable interference induced by PV water extract treatment on the plasma levels of selected metabolism enzymes and precursor molecules of FOLFOX component drugs in colon tumor-bearing mice. Furthermore, the combined use of FOLFOX and PV water extract could result in better anti-tumor and anti-metastasis efficacy in colon tumor-bearing mouse models. Therefore, the beneficial potentials of the combined use of FOLFOX and PV water extract has been scientifically verified, which may support the clinical use in colon cancer management.

## Data Availability

The original contributions presented in the study are included in the article/supplementary material, further inquiries can be directed to the corresponding author.

## References

[B1] Andre T. Boni C. Navarro M. Tabernero J. Hickish T. Topham C. (2009). Improved overall survival with oxaliplatin, fluorouracil, and leucovorin as adjuvant treatment in stage II or III colon cancer in the MOSAIC trial. J. Clin. Oncol. 27, 3109–3116. 10.1200/JCO.2008.20.6771 19451431

[B2] Bray F. Laversanne M. Sung H. Ferlay J. Siegel R. L. Soerjomataram I. (2024). Global cancer statistics 2022: GLOBOCAN estimates of incidence and mortality worldwide for 36 cancers in 185 countries. CA Cancer J. Clin. 74, 229–263. 10.3322/caac.21834 38572751

[B3] Burz C. Berindan-Neagoe I. B. Balacescu O. Tanaselia C. Ursu M. Gog A. (2009). Clinical and pharmacokinetics study of oxaliplatin in colon cancer patients. J. Gastrointestin Liver Dis. 18, 39–43. 19337632

[B4] Catanzaro D. Filippini R. Vianello C. Carrara M. Ragazzi E. Montopoli M. (2016). Chlorogenic acid interaction with cisplatin and oxaliplatin: studies in cervical carcinoma cells. Nat. Prod. Commun. 11, 499–502. 27396204

[B5] Center for Drug Evaluation and Research (2005). Guidance for industry: estimating the maximum safe starting dose in initial clinical trials for therapeutics in adult healthy volunteers. Available online at: https://www.fda.gov/media/72309/download (Accessed November 21, 2025).

[B6] Cervantes A. Adam R. Rosello S. Arnold D. Normanno N. Taieb J. (2023). Metastatic colorectal cancer: ESMO clinical practice guideline for diagnosis, treatment and follow-up. Ann. Oncol. 34, 10–32. 10.1016/j.annonc.2022.10.003 36307056

[B7] Chen P. Ni W. Xie T. Sui X. (2019). Meta-analysis of 5-fluorouracil-based chemotherapy combined with traditional Chinese medicines for colorectal cancer treatment. Integr. Cancer Ther. 18, 1534735419828824. 10.1177/1534735419828824 30791729 PMC7242800

[B8] Chen M. X. Fang C. W. Yang Q. S. Jin J. K. Ding Z. F. Li L. Y. (2020). Pharmacognosic identification of six medicinal plants of *patrinia* . J. Anhui Univ. Chin. Med. 39, 78–81.

[B9] Cho H. K. Lee E. S. Lee J. W. Park J. K. Kang J. H. Lee K. S. (2006). Clinical pharmacokinetics of oxaliplatin and 5-fluorouracil administered in combination with leucovorin in Korean patients with advanced colorectal cancer. J. Cancer Res. Clin. Oncol. 132, 320–326. 10.1007/s00432-005-0072-6 16395593 PMC12161070

[B10] Delord J. P. Umlil A. Guimbaud R. Gregoire N. Lafont T. Canal P. (2003). Population pharmacokinetics of oxaliplatin. Cancer Chemother. Pharmacol. 51, 127–131. 10.1007/s00280-002-0550-3 12647013

[B11] Deyme L. Barbolosi D. Gattacceca F. (2019). Population pharmacokinetics of FOLFIRINOX: a review of studies and parameters. Cancer Chemother. Pharmacol. 83, 27–42. 10.1007/s00280-018-3722-5 30446786

[B12] Dhelens C. Bonadona A. Thomas F. Chapuis C. Potton L. Marsili S. (2016). Lethal 5-fluorouracil toxicity in a colorectal patient with severe dihydropyrimidine dehydrogenase (DPD) deficiency. Int. J. Colorectal Dis. 31, 699–701. 10.1007/s00384-015-2191-0 25796495

[B13] Di Francesco A. M. Ruggiero A. Riccardi R. (2002). Cellular and molecular aspects of drugs of the future: oxaliplatin. Cell Mol. Life Sci. 59, 1914–1927. 10.1007/pl00012514 12530522 PMC11337462

[B14] Ducker G. S. Ghergurovich J. M. Mainolfi N. Suri V. Jeong S. K. Hsin-Jung Li S. (2017). Human SHMT inhibitors reveal defective glycine import as a targetable metabolic vulnerability of diffuse large B-cell lymphoma. Proc. Natl. Acad. Sci. U. S. A. 114, 11404–11409. 10.1073/pnas.1706617114 29073064 PMC5664509

[B15] Gong L. Zou W. Zheng K. Shi B. Liu M. (2021). The herba patriniae (caprifoliaceae): a review on traditional uses, phytochemistry, pharmacology and quality control. J. Ethnopharmacol. 265, 113264. 10.1016/j.jep.2020.113264 32846192 PMC7443212

[B16] Greiner P. O. Zittoun J. Marquet J. Cheron J. M. (1989). Pharmacokinetics of (-)-folinic acid after oral and intravenous administration of the racemate. Br. J. Clin. Pharmacol. 28, 289–295. 10.1111/j.1365-2125.1989.tb05429.x 2789922 PMC1379947

[B17] Guan Y. Kraus S. G. Quaney M. J. Daniels M. A. Mitchem J. B. Teixeiro E. (2020). FOLFOX chemotherapy ameliorates CD8 T lymphocyte exhaustion and enhances checkpoint blockade efficacy in colorectal cancer. Front. Oncol. 10, 586. 10.3389/fonc.2020.00586 32391270 PMC7190812

[B18] Hao H. Zhang Y. Hu X. Guo W. Yang C. Wang J. (2023). Cocrystallization of 5-fluorouracil with gallic acid: a novel 5-fluorouracil cocrystal displaying synergistic anti-tumor activity both in oral and intraperitoneal injection administration. Eur. J. Pharm. Biopharm. 187, 12–23. 10.1016/j.ejpb.2023.04.001 37031731

[B19] He X. Luan F. Zhao Z. Ning N. Li M. Jin L. (2017). The genus *patrinia*: a review of traditional uses, phytochemical and pharmacological studies. Am. J. Chin. Med. 45, 637–666. 10.1142/S0192415X17500379 28595500

[B20] Heggie G. D. Sommadossi J. P. Cross D. S. Huster W. J. Diasio R. B. (1987). Clinical pharmacokinetics of 5-fluorouracil and its metabolites in plasma, urine, and bile. Cancer Res. 47, 2203–2206. 3829006

[B21] Huang S. Z. Liu W. Y. Huang Y. Shen A. L. Liu L. Y. Peng J. (2019). *Patrinia scabiosaefolia* inhibits growth of 5-FU-resistant colorectal carcinoma cells via induction of apoptosis and suppression of AKT pathway. Chin. J. Integr. Med. 25, 116–121. 10.1007/s11655-018-3002-6 29948597

[B22] Jiang N. N. Yue G. G. L. Li P. Ye Y. S. Gomes A. J. Kwok F. H. F. (2022). Discovery of dearomatized isoprenylated acylphloroglucinols with colon tumor suppressive activities in mice via inhibiting NFkappaB-FAT1-PDCD4 signaling activation. Eur. J. Med. Chem. 239, 114532. 10.1016/j.ejmech.2022.114532 35749988

[B23] Johnson M. R. Diasio R. B. (2001). Importance of dihydropyrimidine dehydrogenase (DPD) deficiency in patients exhibiting toxicity following treatment with 5-fluorouracil. Adv. Enzyme Regul. 41, 151–157. 10.1016/s0065-2571(00)00011-x 11384742

[B24] Li M. Song L. H. Yue G. G. Lee J. K. Zhao L. M. Li L. (2017). Bigelovin triggered apoptosis in colorectal cancer *in vitro* and *in vivo* via upregulating death receptor 5 and reactive oxidative species. Sci. Rep. 7, 42176. 10.1038/srep42176 28181527 PMC5299840

[B25] Li M. Yue G. G. L. Tsui S. K. Fung K. P. Lau C. B. S. (2018). Turmeric extract, with absorbable curcumin, has potent anti-metastatic effect *in vitro* and *in vivo* . Phytomedicine 46, 131–141. 10.1016/j.phymed.2018.03.065 30097113

[B26] Li X. C. Wang S. Yang X. X. Li T. J. Gu J. X. Zhao L. (2023). *Patrinia villosa* treat colorectal cancer by activating PI3K/Akt signaling pathway. J. Ethnopharmacol. 309, 116264. 10.1016/j.jep.2023.116264 36868440

[B27] Lim S. C. Lee T. B. Han S. I. (2024). Caffeic acid enhances anticancer drug-induced apoptosis in acid-adapted HCT116 colon cancer cells. Anticancer Res. 44, 2587–2595. 10.21873/anticanres.17064 38821580

[B28] Lin C. H. Jiang W. P. Itokazu N. Huang G. J. (2025). Chlorogenic acid attenuates 5-fluorouracil-induced intestinal mucositis in mice through SIRT1 signaling-mediated oxidative stress and inflammatory pathways. Biomed. Pharmacother. 186, 117982. 10.1016/j.biopha.2025.117982 40106967

[B29] Maindrault-Goebel F. De Gramont A. Louvet C. Andre T. Carola E. Mabro M. (2001). High-dose intensity oxaliplatin added to the simplified bimonthly leucovorin and 5-fluorouracil regimen as second-line therapy for metastatic colorectal cancer (FOLFOX 7). Eur. J. Cancer 37, 1000–1005. 10.1016/s0959-8049(01)00068-5 11334725

[B30] Mattison L. K. Soong R. Diasio R. B. (2002). Implications of dihydropyrimidine dehydrogenase on 5-fluorouracil pharmacogenetics and pharmacogenomics. Pharmacogenomics 3, 485–492. 10.1517/14622416.3.4.485 12164772

[B31] Miura T. Suzuki N. Nakamura J. Yamada S. Miura T. Yanagi M. (2010). Hepatocellular carcinoma, with portal thrombus after viral eradication, disappeared by 5-fluorouracil and interferon. World J. Hepatol. 2, 416–418. 10.4254/wjh.v2.i11.416 21173911 PMC3004036

[B32] Motamedi Z. Amini S. A. Raeisi E. Lemoigne Y. Heidarian E. (2020). Combined effects of protocatechuic acid and 5-fluorouracil on p53 gene expression and apoptosis in gastric adenocarcinoma cells. Turk J. Pharm. Sci. 17, 578–585. 10.4274/tjps.galenos.2019.69335 33389946 PMC7786068

[B33] Pourghasemian M. Danandeh Mehr A. Molaei M. Habibzadeh A. (2020). Outcome of FOLFOX and modified DCF chemotherapy regimen in patients with advanced gastric adenocarcinoma. Asian Pac J. Cancer Prev. 21, 2337–2341. 10.31557/APJCP.2020.21.8.2337 32856863 PMC7771915

[B34] Robinson S. M. Mann J. Manas D. M. Mann D. A. White S. A. (2013). An experimental study to identify the potential role of pharmacogenomics in determining the occurrence of oxaliplatin-induced liver injury. HPB Oxf. 15, 581–587. 10.1111/hpb.12010 23458185 PMC3731578

[B35] Sharma V. Gupta S. K. Verma M. (2019). Dihydropyrimidine dehydrogenase in the metabolism of the anticancer drugs. Cancer Chemother. Pharmacol. 84, 1157–1166. 10.1007/s00280-019-03936-w 31482228

[B36] Theile D. Grebhardt S. Haefeli W. E. Weiss J. (2009). Involvement of drug transporters in the synergistic action of FOLFOX combination chemotherapy. Biochem. Pharmacol. 78, 1366–1373. 10.1016/j.bcp.2009.07.006 19622348

[B37] Van Kuilenburg A. B. Hausler P. Schalhorn A. Tanck M. W. Proost J. H. Terborg C. (2012). Evaluation of 5-fluorouracil pharmacokinetics in cancer patients with a c.1905+1G>A mutation in DPYD by means of a bayesian limited sampling strategy. Clin. Pharmacokinet. 51, 163–174. 10.1007/BF03257473 22339448

[B38] Wang S. Lai X. Deng Y. Song Y. (2020). Correlation between mouse age and human age in anti-tumor research: significance and method establishment. Life Sci. 242, 117242. 10.1016/j.lfs.2019.117242 31891723

[B39] Wang J. Wang X. Ma X. Xu B. Chen L. Chen C. (2022). Therapeutic effect of *Patrinia villosa* on TNBS-induced ulcerative colitis via metabolism, vitamin D receptor and NF-kappaB signaling pathways. J. Ethnopharmacol. 288, 114989. 10.1016/j.jep.2022.114989 35032589

[B40] Wong K. H. Zheng T. Yue G. G. Li M. C. Wu H. Y. Tong M. H. (2024). A systematic approach for authentication of medicinal *patrinia* species using an integration of morphological, chemical and molecular methods. Sci. Rep. 14, 6566. 10.1038/s41598-024-57115-w 38503940 PMC10951358

[B41] Xia L. Zhang B. Yan Q. Ruan S. (2018). Effects of saponins of *Patrinia villosa* against invasion and metastasis in colorectal cancer cell through NF-kappaB signaling pathway and EMT. Biochem. Biophys. Res. Commun. 503, 2152–2159. 10.1016/j.bbrc.2018.08.005 30119890

[B42] Xu H. Yang T. Liu X. Tian Y. Chen X. Yuan R. (2016). Luteolin synergizes the antitumor effects of 5-fluorouracil against human hepatocellular carcinoma cells through apoptosis induction and metabolism. Life Sci. 144, 138–147. 10.1016/j.lfs.2015.12.002 26656468

[B43] Yan Y. Li J. Han J. Hou N. Song Y. Dong L. (2015). Chlorogenic acid enhances the effects of 5-fluorouracil in human hepatocellular carcinoma cells through the inhibition of extracellular signal-regulated kinases. Anticancer Drugs 26, 540–546. 10.1097/CAD.0000000000000218 25714249 PMC4415958

[B44] Yan X. J. Wei L. Ying Z. Ning C. Ying X. Jian W. (2016). A new biphenyl neolignan from leaves of *Patrinia villosa* (thunb.) juss. Pharmacogn. Mag. 12, 1–3. 10.4103/0973-1296.175988 27019553 PMC4787329

[B45] Yang H. Cheung M. K. Yue G. G. L. Leung P. C. Wong C. K. Lau C. B. S. (2021). Integrated network pharmacology analysis and *in vitro* validation revealed the potential active components and underlying mechanistic pathways of herba patriniae in colorectal cancer. Molecules 26, 6032. 10.3390/molecules26196032 34641576 PMC8513027

[B46] Yang H. Yue G. G. L. Yuen K. K. Gao S. Leung P. C. Wong C. K. (2023). Mechanistic insights into the anti-tumor and anti-metastatic effects of *Patrinia villosa* water extract in colon cancer via modulation of TGF-beta R1-smad2/3-E-cadherin and FAK-RhoA-cofilin pathways. Phytomedicine 117, 154900. 10.1016/j.phymed.2023.154900 37269754

[B47] Zhang T. Li Q. Li K. Li Y. Li J. Wang G. (2008). Antitumor effects of saponin extract from *Patrinia villosa* (thunb.) juss on mice bearing U14 cervical cancer. Phytother. Res. 22, 640–645. 10.1002/ptr.2354 18350512

